# “No BLT”: The Rise and Risk of Acronyms in the Medical Record

**DOI:** 10.1007/s10730-026-09581-0

**Published:** 2026-02-25

**Authors:** Isha Harshe, Rebecca Soistmann, Kenneth Goodman

**Affiliations:** Miller School of Medicine, University of Miami, Miami, USA

**Keywords:** Acronym · Abbreviation · Communication · Documentation · Electronic Health Record

## Abstract

BLT, SSCP, WHOL—the use of acronyms has expanded, comprising large portions of most clinicians’ notes. We present examples of acronyms found in medical records at our institutions and collected as part of an initiative among three ethics committees to demonstrate not only their widespread use, but also the breadth of the types of acronyms used in the medical record. Acronyms have traditionally been used in medical charting to increase efficiency. However, acronyms can impede patient care because they are prone to misinterpretations and misunderstandings between medical professionals. Such misunderstandings have led to incorrect pharmaceutical dosage administration and misdiagnoses. With patients gaining access to their medical records under the Cures Act in 2020, clinicians must also consider the readability of their notes not only for colleagues, but also patients. We provide four different solutions to help mitigate the use of acronyms in medical records while balancing clinician interests in efficiency: creating and implementing “do not use” lists for commonly misunderstood acronyms, leading educational sessions and audits to ensure clinical care team members adhere to “do not use” lists and “approved acronyms” lists, implementing an Electronic Health Record with functionality to create “user dictionaries” in which typed acronyms automatically become full phrases, and seeking feedback from care team members and patients on whether notes have become more “readable” after implementation of initial strategies. Though the use of acronyms may continue to be a feature of medical communication, we hope to encourage the reduction and then judicious use of acronyms.

## Introduction

Medical record: “Past medical history significant for PAD, CKD, HTN, HLD, DM, CAD w/ prior CABG 20yrs ago (LIMA-LAD and known occluded SVG to RCA and SVG to Cx), prior PCIs”.

English: “This patient’s history is significant for peripheral arterial disease, chronic kidney disease, hypertension, hyperlipidemia, diabetes mellitus, coronary artery disease with prior coronary artery bypass graft 20 years ago (left internal mammary artery—left anterior descending and known occluded saphenous venous graft to right coronary artery and saphenous venous graft to circumflex), prior percutaneous coronary interventions.”

The line quoted above, a sentence from a physician’s progress note, contains three abbreviations, twelve acronyms (one a “pseudo-acronym”) and 14 words. It is difficult to understand. Contemporary electronic health records are littered with acronyms, many of which impede clear communication and so invite miscommunication—which, in turn, can foster medical error.

Clear and accurate documentation of clinical encounters is necessary for patient care. Poor documentation impedes care by obscuring or omitting key elements in patients’ lives and medical histories. This makes it difficult for colleagues to understand those histories and undermines contemporary efforts to include patients as participants in their own care. If a medical record and patient summary say, for instance, “No BLT,” as encountered in one progress note, patients and non-orthopedic colleagues may interpret that as limitations on the patient’s diet when the orthopedic surgeon simply wants her patient to refrain from bending, lifting, and twisting.

The excessive use of acronyms and abbreviations can corrupt clinical documentation. In hospitals and multidisciplinary clinics, reliance on acronyms can also constitute a kind of professional discourtesy when colleagues from one specialty assume that generalists and colleagues from other specialties will be familiar with domain-specific acronyms: when one does not recognize an acronym, one needs to look it up. Moreover, several commonly used acronyms are ambiguous and point to more than one diagnosis, condition or intervention—some wholly unrelated to others. The challenge is acute when a hospital team includes social workers, clergy, philosophers and others: for many such, most acronyms are often occult.

To illustrate the rampant use of acronyms in the electronic health record, and the ethical implications of this phenomenon, we assembled an initiative called the “Acronym Mitigation Project,” or “AMP” for short. This initiative was a recurring agenda item at three different hospital ethics committees affiliated with our institutions, which included the initiative on meeting agendas; we asked physician and nursing colleagues to share unfamiliar acronyms they encountered in clinical notes. These acronyms are compiled in Table 1.

**Table 1:** List of acronyms found in medical records and their intended meanings

Acronym	Meaning
AAO	Alert, Awake, and Oriented
AMS	Altered Mental Status
ANCA	Anti-Neutrophil Cytoplasmic Antibody
ATFL	Anterior Talofibular Ligament
AVCOOBTC	*Unknown*
BIBEMS	Brought In By Emergency Medical Services
BOO	Bladder Outlet Obstruction
BPD	Borderline Personality Disorder
BRBPR	Bright Red Blood Per Rectum
CAP	Community Acquired Pneumonia
CDI	Clinical Documentation Improvement
CKD	Chronic Kidney Disease
CV	Cardiovascular
EMU	Epilepsy Monitoring Unit
ESA	Erythropoietin- Stimulating Agents
ESRD	End Stage Renal Disease
ESUS	Embolic Stroke of Unspecified Source
FGR	Fetal Growth Restriction
GON	Glaucomatous Optic Neuropathy
HLP	Hyperkeratosis Lenticularis Perstans
IDEM	Intradural Extra-Medullary
IMN	Intramedullary Nailing
JVD	Jugular Venous Distention
LAA	Left Atrial Appendage
LKW	Last Known Well
M/R/G	Murmurs, Rubs, Gallops
MSK	Musculoskeletal
NIPT	Non-invasive Prenatal Testing
NAD	No Acute Distress
NIV	Non-Invasive Ventilation
NPH	Normal Pressure Hydrocephalus
NRFHT	Non-Reassuring Fetal Heart Tones
OTTF	Ortho Trauma To Follow
PERRLA	Pupils Equal, Round, React to Light and Accommodation
PHBC	Pedestrian Hit By Car
PMH	Past Medical History
PNES	Psychogenic Non-Epileptic Seizures
POD	Progression of Disease
POTS	Postural Orthostatic Tachycardic Syndrome
PMSR	Post-Mortem Sperm Retrieval
RCC	Renal Cell Carcinoma
RIJ	Right Internal Jugular
RRR	Regular Rate & Rhythm
SAH	Subarachnoid Hemorrhage
SGA	Small for Gestational Age
SOB	Shortness of Breath
SNF	Skilled Nursing Facility
SSCP	Substernal Chest Pain
SI/HI/AVH	Suicidal Ideation, Homicidal Ideation, Auditory/Visual Hallucinations
TTS	Tuesday, Thursday, Saturday
VGAM	Vein of Galen Arterial Malformation
VSD	Ventricular Septal Defect
WOALS	*Unknown*
WHOL	Worst Headache of Life
WOB	Work of Breathing

## Acronyms in Medicine

Acronyms have been found in 99.1% of clinical record notes (Lee et al., 2017) and are routinely used to save time when documenting patient encounters, interventions and medical histories. One study found that acronyms comprised 30–50% of the words in a medical admission note at their urban academic medical center (Grossman et al., 2018). This leads to an “alphabet soup” phenomenon where a patient note can become almost indecipherable. Table 2 highlights some examples found at our institutions.

**Table 2:** Examples of “alphabet soup” phenomenon in clinical documentation

“PMH of AMA+ PRBC, CREST, IDA, EBV+ sent to ED for SBP, sp EGD EVB, ?EVL. AHRF, then extubated sent to VIPU for EOL POC”
“s/p ORIF with syndesmotic repair was sent to the ED with c/o L foot swelling to r/o DVT”
“history of HTN, HLD, MDD/GAD, history of AUD c/b AWS seizures in remission, history of acute pancreatitis and CAD with recent LHC and stent placement on DAPT.”
“Hx of SLE complicated by lupus nephritis on HD TTS, HFREF 10–15% (NICM 22/2 cyclophosphamide tox)”
“P1314 s/p emergent rLTCD at 25.5 wks for NRFHT”

Though efficiency, speed, and convenience are leading reasons for acronym use, some underlying sociological factors might contribute to the problem as well. For instance, acronym use may be perceived as demonstrating knowledge or competence in a specific specialty. It creates a special language and social hierarchy within the profession in which the use of acronyms is seen as “savvy” (Parvaiz et al., 2008). If worthiness to join an exalted club entails intimate knowledge of the nomenclature, acronyms become part of the initiation. Contrarily, many good physicians have no such view or intent, and defend acronym use as a legitimate buffer against some forms of documentation burnout.

The use of acronyms in this context is akin to the earlier impact of physicians’ illegible handwriting, which caused miscommunication leading to inappropriate patient management and even patient deaths (Ariaga et al., 2023; Sokol & Hettige, 2006). Poor handwriting among physicians had been so widespread that it was comically considered by some a requirement for becoming a doctor. Associating a practice with a specific group of people creates a sense of “otherness,” a secret language for physicians, thus perpetuating a professional and social hierarchy (Laderman, 2006). The electronic health record has nearly eliminated the use of handwritten notes in health records; the use of acronyms is unabated.

### How Acronyms Jeopardize Patient Care

Though acronyms and abbreviations might be useful for the author of a medical report, those who must interpret the report face the real challenge. Medicine is an interdisciplinary field deeply reliant on collaboration, and successful teamwork demands clear communication. Numerous studies have been conducted in which physicians at different levels of training and other health professionals were given acronyms commonly used in their own or a different specialty to interpret (Awan et al., 2016; Hamiel et al., 2018; Jayatilake & Oyibo, 2023; Parvaiz et al., 2008; Sheppard et al., 2008; Sherriff et al., 2017; Sinha et al., 2011; Vale et al., 2017). Across a wide variety of specialties, no participant in these studies could identify all the acronyms accurately. One study showed that oncologists scored the highest (71.6%) amongst different specialty groups when interpreting notes from oncology, surgery, gynecology, pediatrics, and internal medicine. On the other hand, pediatricians scored the lowest (55.4%) when given the same set of acronyms (Sherriff et al., 2017). These results demonstrate that the varying amount of expertise, exposure, and overlap amongst specialties also affect the ability of different specialists to interpret acronyms found in other specialties. Another study found that even within their own specialty, only 57.24% of the orthopedic surgeons could accurately interpret acronyms found in orthopedic surgery notes (Parvaiz et al., 2008). The electronic health record is not only regularly referred to by physicians and other clinicians for patient care but also allied health professionals such as nurses and dieticians. When comparing the performance of different professionals in deciphering a list of 30 acronyms taken from surgical patient notes, dietitians scored the lowest with an overall correct response of 21%. Nurses scored 30%, while first-year doctors scored the highest at 57% (Sinha et al., 2011). Though no group of healthcare professionals scored optimally, the disparities between each group demonstrate that acronym use in medical records impedes communication between not only different specialties, but also different types of healthcare professionals.

Others’ use of unfamiliar acronyms in medical records is frustrating for medical professionals. One study reported that “93% of respondents claimed to get ‘irritated’ or ‘very irritated’ when they encounter an abbreviation they are not familiar with” (Hamiel et al., 2018). Many clinicians rely on using context clues or look up acronyms on search engines to find their meanings, Hamiel et al. (2018) which can lead to misinterpretation if the wrong meaning is accepted. Many acronyms that have multiple meanings can be easily misattributed in patient notes (Table 3). In conjunction with copying and pasting from earlier notes—another well-documented inducement to medical error—misinterpreted acronyms can be perpetuated for years. An example of this includes a patient who was thought to have cerebral palsy for 4 years when the initial “CP” acronym was intended to stand for “cleft palate” (Kuhn, 2007). Clinicians sometimes also skip over an acronym if they do not readily understand it, thus ignoring potentially critical information about the patient (Parry et al., 2023).

Different languages have different acronyms for the same concept, potentially complicating international communication and interpretation of literature. For example, AIDS, Acquired Immunodeficiency Syndrome, is referred to as SIDA in French (Syndrome Immuno-Deficitaire Acquis) (Armocida et al., 2024). In areas with health professionals from different countries, there may be miscommunication because people are accustomed to the acronyms used in their training country (Haseeb et al., 2016).

Acronyms indisputably cause misunderstandings in medical communication. How these misunderstandings translate to medical harm and decreased quality of care for patients may be more challenging to prove, given the number of structural, behavioral, and personal factors that combine to cause them. In the United States from 2004 to 2006, 4.7% of all medical errors, 29,974 incidents in total, reported through the National Medication Error Reporting program were due to misinterpretation of medical abbreviations (Brunetti et al., 2007). Further, the United Kingdom’s National Health Service recorded 384 “never events” from April 2022 to March 2023. The National Health Service defines “never events” as serious, largely preventable patient safety incidents that should not occur if healthcare providers have implemented existing national guidance or safety recommendations. Ineffective communication, including acronym use, was the most-cited behavioral factor driving these never events (NHS England, 2023). Root-cause analyses of patient safety incidents are often lengthy and multifaceted, but acronym use propelling misunderstanding and miscommunication certainly contributes.

**Table 3:** Examples of acronyms and their multiple medical meanings

Acronym	Medical meanings
ABG	Air Bone Gap Arterial Blood Gas
ADH	Anti-diuretic Hormone Atypical Ductal Hyperplasia
AIS	Acute Ischemic Stroke Adenocarcinoma In Situ Alcohol-Insoluble Solids Anal Carcinoma In Situ Androgen-Insensitivity Syndrome Anterior Interosseus Nerve Syndrome Adolescent Idiopathic Scoliosis
AMA	Advanced Maternal Age Anti-mitochondrial antibodies Against Medical Advice American Medical Association
BLT	Bending, Lifting, or Twisting Balanced Ligamentous Tension Bilateral Lung Transplantation Blood Test Bright Light Test Battery of Leukocyte Tests
C/F	Consistent For Capillary-to-fiber Capillary/Fiber Ratio Chills/Fever Coagulation/Flocculation Cystic Fibrosis
CP	Cerebral Palsy Chest Pain Cleft Palate Cardiopulmonary Cicatricial Pemphigoid
CPC	Clinicopathologic Conference Certified Professional Coder Choroid Plexus Cauterization Choroid Plexus Cyst Chronic Passive Congestion Cyclophotocoagulation Child-Pugh C Cirrhosis
**ESA**	Erythropoietin-Stimulating Agents Emotional Support Animal Emergency and Safety Alliance
FOB	Foot of Bed Father of Baby Fiberoptic Bronchoscopy Fecal Occult Blood
HLD	Haloperidol Decanoate Healed Hepatolenticular Degeneration Herniated Lumbar Disc Hippel Lindau Disease Hyperlipidemia Hypersensitivity Lung Disease
LAD	Left Anterior Descending Lymphadenopathy Leukocyte Adhesion Deficiency Left Axis Deviation
LUS	Laparoscopic Ultrasound Lung Ultrasound Score Largescale Unbiased Sequencing Laryngeal Ultrasound Leg Ulcers Laser Ultrasound Lighted Ureteral Stents Longest Uninterrupted Stretch
OM	Organic Matter Osteomyelitis Obtuse and marginal, coronary artery branching from the circumflex coronary artery Otitis media Occipito-Mesencephalic
PeAF	Peak Expiratory Air Flow Persistent Atrial Fibrillation Partial Epilepsy with Auditory Features Permanent Atrial Fibrillation
SLE	Slit Lamp Exam Systemic Lupus Erythematosus
SVD	Spontaneous Vaginal Delivery Structural Valve Deterioration Significant Valvular Disease Spontaneous Vertex Delivery Singular Valve Decomposition Snowflake Vitreoretinal Degeneration Small Vessel Disease Star Volume Distribution Subcortical Vascular Dementia Severe Variable Deceleration Single-Vessel Disease
TDC	Tunneled Dialysis Catheter Thyroglossal Duct Cysts Transitional Dialysis Care
UC	Urgent Care Ulcerative Colitis Urine Culture

Though acronyms may seem efficient for their users, they can lead to an increased burden for readers. Writing an acronym instead of spelling out its component words saves about 20.5 seconds whereas an extra 12–84.5 seconds has been documented as the time needed to decipher an acronym (Zetner et al., 2022). The extra time it takes to understand the acronym may deter others from attempting to understand it.

Acronyms can also undermine patients’ ability to understand their physicians. In 2020, the Office of the National Coordinator for Health Information Technology, now the Assistant Secretary for Technology Policy, approved the Cures Act Final Rule, which requires that patients have access to their electronic health records. The change was justified in part by evidence that such access improves outcomes (Shenkin & Warner, 1973). Access to medical documentation helps patients become informed and stronger advocates for themselves. Acronym use impedes this and undermines physicians as health educators. When patient comprehension of clinical information in medical charts was compared with and without use of common medical acronyms, comprehension performance jumped from 62% to 95% when expanded terms were instead of acronyms (Grossman Liu et al., 2022).

This issue is highly pertinent in a world of rapid adoption of artificial intelligence, especially in health care. Artificial intelligence has many use cases in health care and medicine, and artificial intelligence scribes represent a key area of relevance to acronym use and its mitigation. Using a large language model, artificial intelligence scribes can employ speech recognition during a patient encounter, generate a patient note, write an encounter summary, and draft a follow-up email for the patient, all within minutes. The opportunity available here is tremendous—but such opportunity is accompanied by nontrivial risk. In one case, an artificial intelligence scribe interpreted the phrase “Sciton BBL,” referring to Broad Band Light for facial skin, as “Sciton Brazilian Butt Lift” (Mess et al., 2025). The issues that acronyms pose to medical charting persist even in the face of the newest technology. If large language models for artificial intelligence-based tools are trained using existing medical records, acronyms that have many different meanings could contribute to AI hallucination and error.

## Mitigating Acronym Use in Patient Care

There are several ways to reduce the use of acronyms in clinical documentation. They can be tested to determine if efficiency has been increased and administrative burden reduced.

1. *Create “Do Not Use” Lists of Acronyms*.

Patient safety is paramount when it comes to misinterpreting acronyms. Several solutions have been proposed to mitigate acronym use in medical communication, one of which is widely recognized. The Joint Commission, which certifies health care organizations in part based on quality and outcomes, has published a list of acronyms deemed to be dangerous when used in medical records. The “Do Not Use” list “bans” the use of specific acronyms (Parry et al., 2023; Tariq & Sharma, 2024), for instance using “MS,” which can be interpreted as either morphine sulfate or magnesium sulfate, both of which are commonly used. The Joint Commission also encourages hospitals and other health institutions to create their own “Do Not Use” lists and prohibits acronyms in patient-facing documents such as informed consent forms and discharge instructions (Tariq & Sharma, 2024). Such lists can ensure that the most harmful types of patient care errors that can result from acronym and abbreviation usage, such as incorrect dosages, are prevented.

2. *Create standardized acronym lists by specialty*.

Medical specialties could generate lists of approved acronyms to be used in those specific specialties (Perez et al., 2022; Ramana et al., 2020). However, the development of such lists could prove challenging, as different hospitals and systems have different meanings for the same acronyms. For example, “AA” is listed as “Alcoholics Anonymous” in one Florida hospital’s approved list but as “Acute Appendectomy” in the approved list of a hospital in Idaho. Providers who train and work at different institutions may not realize that the acronym they used at one institution has a completely different meaning at another institution. If specialty-specific societies were to generate “approved acronym lists” to be wholly adopted by all members of that specialty, this may help to decrease variability across institutions.

3. *Educate clinicians on the risks involved with acronym use*.

Several studies document educational efforts that reduced the use of acronyms in medical records (Alshaikh et al., 2013; Haseeb et al., 2016; Horon et al., 2012; Sheppard et al., 2008; Taylor et al., 2007). These initiatives used lists of acceptable and unacceptable acronyms and abbreviations and posted them on desktop screens. One hospital prohibited the use of unsafe abbreviations and hosted educational sessions (Alshaikh et al., 2013). The support of senior clinicians and the use of audits to measure changes in practice is reckoned necessary to ensure success (Sheppard et al., 2008). Inviting clinicians to become engaged with the acronym mitigation initiative rather than subject to it helps create an environment focused on patient safety as a shared responsibility, and such educational sessions provide a safe, collaborative space for learning how to uphold this responsibility.

4. *Automate the elimination of acronyms*.

It is possible to adapt existing auto-correct tools to identify and spell out acronyms in medical records. Some electronic health record vendors enable users to create lists in which they list acronyms and their intended meanings such that whenever they type the acronym and hit space or a punctuation mark, the acronym automatically expands into the full phrase. Indeed, many documentation systems already include “smart text” or “smart phrases” which, when invoked, save the trouble of typing out the full phrase.

It is comparatively straightforward for a team to identify acronyms in general and specialty use. The latter, one ought to hypothesize, would be valued by both patients and clinicians from other specialties (for instance, “TOLAC” for “trial of labor after caesarean”, “BPP” for “biophysical profile” and “AUB” for “abnormal uterine bleeding”, all commonly used in obstetrics and gynecology but unfamiliar to those in other specialties). Clinicians can transform their notes from something that is difficult to interpret into something that can be easily read and understood in all parts of the hospital. Such a function balances both the need for efficiency and ensuring understanding without compromising more time or effort on the part of the person writing the note.

## Limitations of Acronym Mitigation

In an ideal world, the use of acronyms in medical communication—both written and verbal—would be completely eliminated to enhance understanding, teaching, and efficiency across health care teams. However, we would be remiss if we did not acknowledge that this may not be an entirely practical solution given the impact acronyms have already sustained on medical communication. Namely, certain acronyms have come to symbolize the idea they represent, and the full form of such acronyms is limited in our dialogue. Examples of such acronyms are “HIV” or “CT scan.” In these cases, HIV is more easily understood rather than “human immunodeficiency virus” because the acronym has become the colloquial representation of the term. This is where clinician discretion becomes vital in deciding whether an acronym is appropriate to use in medical communication—whereas HIV is commonly known, CMV, which stands for cytomegalovirus, is less prevalent and thus may be lesser known, and should be spelled out. Situations like these present limited circumstances where acronym use may be permissible. Especially in the context of patient access to medical charts under the Cures Act, we recommend a process to consider whether a patient with a non-medical background would be able to understand a given acronym if it were written in the chart. Regardless, perhaps the simplest rule to keep would be, when in doubt, just spell it out.

## Ethics and Acronyms

Duties of veracity, transparency, and fidelity are uncontroversial. It is therefore a mystery that the use of acronyms is so widespread and has been sustained so long. Once one is informed that acronym use impedes understanding by colleagues and patients, which one surely would want to prevent, it seems a clear-cut obligation to refrain from their use. This lesson had been learned, in part, by earlier documentation of the risks enhanced by poor handwriting.

If there is reason to believe that acronyms impede understanding—we show that they do—then it is a straightforward matter to frame a testable hypothesis further to document harms and adverse events caused by the impediment. Such a test would be a worthy undertaking. For now, it should be sufficient to observe that excessive use of acronyms poses under-addressed ethical challenges for clinicians. Indeed, it is a striking gap in the literature on medical communication that bedside struggles over giving bad news are not accompanied by the communication difficulties shaped by the burdens of documentation. That medical records are as useful for billing and insurance as they are for medical care is well known. It is a pity in parallel that we emphasize progress-note thoroughness for billing purposes, without commensurate attention to the needs of others on a team to know exactly what has happened already. The electronic health record serves as a permanent repository of communication between and among health professionals. Acronym use impedes an essential purpose of medical records by propagating misunderstandings that undermine clinician communication, which can impede patient care. Thus, the present state of acronym use requires intervention to avoid patient harm, the virtues of which we assume to be uncontroversial.

Our Acronym Mitigation Project—“AMP” was too good to pass up—engaged three ethics services at two academic medical centers for several years. Colleagues enjoyed sharing examples, even confessing ignorance of newly found acronyms. It is well established that ethics services serve three primary functions: education, policy work, and consultations. We found that special projects, perhaps best regarded here as quality improvement efforts, should be regarded as among the legitimate duties of such services.

## Conclusion

The use of acronyms is as widespread as it is risky. Acronyms hinder communication and diminish understanding. They are a threat to patient safety and undermine the ability of patients to participate in their care. Acronyms are increasingly subspecialized to the point where they may become unrecognizable for providers within and outside a specialty. Several strategies are available to address problems caused by using acronyms. At the least, discretion is required for acronym use. Clinicians should strive to write patient care notes understandable to anyone without a healthcare background. Forming a more conscientious approach to using acronyms in medical communication can help lead to safer and more effective patient care.

## Data Availability

No datasets were generated or analysed during the current study.

## References

[CR1] Alshaikh, M., Mayet, A., Adam, M., Ahmed, Y., & Aljadhey, H. (2013). Intervention to reduce the use of unsafe abbreviations in a teaching hospital. *Saudi Pharmaceutical Journal (SPJ): The Official Publication of the Saudi Pharmaceutical Society*, *21*(3), 277–280. 10.1016/j.jsps.2012.10.006 23960844 10.1016/j.jsps.2012.10.006 PMC3745070

[CR2] Ariaga, A., Balzan, D., Falzon, S., & Sultana, J. (2023). A scoping review of legibility of hand-written prescriptions and drug-orders: The writing on the wall. *Expert Review of Clinical Pharmacology*, *16*(7), 617–621. 10.1080/17512433.2023.2223972 37308401 10.1080/17512433.2023.2223972

[CR3] Armocida, E., Masciangelo, G., & Natale, G. (2024). Medical eponyms versus acronyms: what medical terminology is most beneficial to learn? A question of goals. *Postgraduate Medical Journal*. 10.1093/postmj/qgae059 10.1093/postmj/qgae059 38710510

[CR4] Awan, S., Abid, S., Tariq, M., Zubairi, A. B., Kamal, A., Arshad, S., Masood, Q., Kashif, W., & Hamid, S. (2016). Use of medical abbreviations and acronyms: Knowledge among medical students and postgraduates. *Postgraduate Medical Journal*, *92*(1094), 721–725. 10.1136/postgradmedj-2016-134086 27281817 10.1136/postgradmedj-2016-134086

[CR5] Brunetti, L., Santell, J. P., & Hicks, R. W. (2007). The impact of abbreviations on patient safety. *Joint Commission journal on quality and patient safety*, *33*(9), 576–583. 10.1016/s1553-7250(07)33062-6 17915532 10.1016/s1553-7250(07)33062-6

[CR7] Grossman, L. V., Mitchell, E. G., Hripcsak, G., Weng, C., & Vawdrey, D. K. (2018). A method for harmonization of clinical abbreviation and acronym sense inventories. *Journal of Biomedical Informatics*, *88*, 62–69. 10.1016/j.jbi.2018.11.004 30414475 10.1016/j.jbi.2018.11.004 PMC6474250

[CR6] Grossman Liu, L., Russell, D., Reading Turchioe, M., Myers, A. C., Vawdrey, D. K., & Masterson Creber, R. M. (2022). Effect of expansion of abbreviations and acronyms on patient comprehension of their health records: A randomized clinical trial. *JAMA Network Open*, *5*(5), e2212320. 10.1001/jamanetworkopen.2022.12320 35560053 10.1001/jamanetworkopen.2022.12320 PMC9107024

[CR8] Hamiel, U., Hecht, I., Nemet, A., Pe’er, L., Man, V., Hilely, A., & Achiron, A. (2018). Frequency, comprehension and attitudes of physicians towards abbreviations in the medical record. *Postgraduate Medical Journal*, *94*(1111), 254–258. 10.1136/postgradmedj-2017-135515 29540451 10.1136/postgradmedj-2017-135515

[CR9] Haseeb, A., Winit-Watjana, W., Bakhsh, A. R., Elrggal, M. E., Hadi, M. A., Mously, A. A., Gadibalban, A. Z., Al-Ibraheem, B. F., Almubark, R. A., Ekram, R. A., & Khan, T. M. (2016). Effectiveness of a pharmacist-led educational intervention to reduce the use of high-risk abbreviations in an acute care setting in Saudi Arabia: a quasi-experimental study. *British Medical Journal Open*, *6*(6), e011401. 10.1136/bmjopen-2016-011401 10.1136/bmjopen-2016-011401 PMC4916617 27311911

[CR10] Horon, K., Hayek, K., & Montgomery, C. (2012). Prohibited abbreviations: Seeking to educate, not enforce. *Canadian Journal of Hospital Pharmacy*, *65*(4), 294–299. 10.4212/cjhp.v65i4.1160 22919107 10.4212/cjhp.v65i4.1160 PMC3420852

[CR11] Jayatilake, D. C., & Oyibo, S. O. (2023). Interpretation and misinterpretation of medical abbreviations found in patient medical records: A cross-sectional survey. *Cureus*, *15*(9), e44735. 10.7759/cureus.44735 37674765 10.7759/cureus.44735 PMC10479966

[CR12] Kuhn, I. F. (2007). Abbreviations and acronyms in healthcare: When shorter isn’t sweeter. *Pediatric Nursing*, *33*(5), 392–398. 18041327

[CR13] Laderman, G. (2006). The cult of doctors: Harvey Cushing and the religious culture of modern medicine. *Journal of Religion and Health*, *45*, 533–548.

[CR14] Lee, E. H., Patel, J. P., & Fortin, A. H. (2017). Patient-centric medical notes: Identifying areas for improvement in the age of open medical records. *Patient Education and Counseling*, *100*(8), 1608–1611. 10.1016/j.pec.2017.02.018 28242141 10.1016/j.pec.2017.02.018

[CR15] Mess, S. A., Mackey, A. J., & Yarowsky, D. E. (2025). Artificial intelligence scribe and large language model technology in healthcare documentation: Advantages, limitations, and recommendations. *Plastic and Reconstructive Surgery – Global Open*, *13*(1), e6450. 10.1097/gox.0000000000006450 39823022 10.1097/GOX.0000000000006450 PMC11737491

[CR16] NHS England. (2023). Provisional publication of never events reported as occurring between 1 April and 31 March 2023. https://www.england.nhs.uk/wp-content/uploads/2023/05/provisional-publication-never-events-1-april-31-march-23.pdf

[CR17] Parry, D., Odedra, A., Fagbohun, M., Oeppen, R. S., Davidson, M., & Brennan, P. A. (2023). Abbreviation use decreases effective clinical communication and can compromise patient safety. *The British Journal of Oral & Maxillofacial Surgery*, *61*(8), 509–513. 10.1016/j.bjoms.2023.07.004 37563053 10.1016/j.bjoms.2023.07.004

[CR18] Parvaiz, M. A., Subramanian, A., & Kendall, N. S. (2008). The use of abbreviations in medical records in a multidisciplinary world–an imminent disaster. *Communications Medicine*, *5*(1), 25–33. 10.1558/cam.v5i1.25 10.1558/cam.v5i1.25 19363877

[CR19] Perez, Z. G., Zafar, M. A., Ziganshin, B. A., & Elefteriades, J. A. (2022). Toward standard abbreviations and acronyms for use in articles on aortic disease. *Journal of Thoracic and Cardiovascular Surgery Open*, *10*, 34–38. 10.1016/j.xjon.2022.04.010 36004246 10.1016/j.xjon.2022.04.010 PMC9390674

[CR20] Ramana, B., Sinha, R., Jacob, B., Towfigh, S., & Rosin, D. (2020). Acronyms use in abdominal wall reconstruction: Introduction to a new language. *World Journal of Surgery*, *44*(1), 78–83. 10.1007/s00268-019-05221-6 31602519 10.1007/s00268-019-05221-6

[CR21] Shenkin, B. N., & Warner, D. C. (1973). Giving the patient his medical record: a proposal to improve the system. *New England Journal of Medicine*, *289*(13), 688–692. 10.1056/nejm197309272891311 4727972 10.1056/NEJM197309272891311

[CR22] Sheppard, J. E., Weidner, L. C., Zakai, S., Fountain-Polley, S., & Williams, J. (2008). Ambiguous abbreviations: An audit of abbreviations in paediatric note keeping. *Archives of Disease in Childhood*, *93*(3), 204–206. 10.1136/adc.2007.128132 17986605 10.1136/adc.2007.128132

[CR23] Sherriff, A., Ally, H., Mahomed, W., Rae, H., Schanknecht, R., Sealanyane, S., & Joubert, G. (2017). The understanding of medical abbreviations across different medical departments in a South African hospital setting. *Communications Medicine*, *14*(1), 15–24. 10.1558/cam.30947 10.1558/cam.30947 29957898

[CR24] Sinha, S., McDermott, F., Srinivas, G., & Houghton, P. W. (2011). Use of abbreviations by healthcare professionals: What is the way forward? *Postgraduate Medical Journal*, *87*(1029), 450–452. 10.1136/pgmj.2010.097394 21459778 10.1136/pgmj.2010.097394

[CR25] Sokol, D. K., & Hettige, S. (2006). Poor handwriting remains a significant problem in medicine. *The Journal of the Royal Society of Medicine*, *99*(12), 645–646. 10.1177/014107680609901219 17139073 10.1258/jrsm.99.12.645 PMC1676338

[CR26] Tariq, R. A., & Sharma, S. (2024). *Inappropriate medical abbreviations*. StatPearls. 30085548

[CR27] Taylor, S. E., Chu, M. T. Y., Haack, L. A., McGrath, A., & To, T. P. (2007). An intervention to reduce the use of error-prone prescribing abbreviations in the emergency department. *Journal of Pharmacy Practice and Research*, *37*(3), 214–216. 10.1002/j.2055-2335.2007.tb00747.x

[CR28] Vale, S. M., Koenig, K., Ailor, S. K., & Martin, K. (2017). Knowledge of dermatologic abbreviations: A survey of patients and physicians who are not dermatologists. *Journal of the American Academy of Dermatology*, *76*(2), 362–364. 10.1016/j.jaad.2016.08.050 28089005 10.1016/j.jaad.2016.08.050

[CR29] Zetner, D. B., Zetner, D. B., Glerup, S. B., Hjortkær, H., Gullach, A., Andersen, K., Overgaard, D. L., & Andresen, K. (2022). Christmas article: Brevity is not to the point—a study of abbreviations. *Ugeskrift For Laeger*, *184*(50), V80110. 36510812

